# Complete genome sequence of *Denitrovibrio acetiphilus* type strain (N2460^T^)

**DOI:** 10.4056/sigs.892105

**Published:** 2010-06-15

**Authors:** Hajnalka Kiss, Elke Lang, Alla Lapidus, Alex Copeland, Matt Nolan, Tijana Glavina Del Rio, Feng Chen, Susan Lucas, Hope Tice, Jan-Fang Cheng, Cliff Han, Lynne Goodwin, Sam Pitluck, Konstantinos Liolios, Amrita Pati, Natalia Ivanova, Konstantinos Mavromatis, Amy Chen, Krishna Palaniappan, Miriam Land, Loren Hauser, Yun-Juan Chang, Cynthia D. Jeffries, John C. Detter, Thomas Brettin, Stefan Spring, Manfred Rohde, Markus Göker, Tanja Woyke, James Bristow, Jonathan A. Eisen, Victor Markowitz, Philip Hugenholtz, Nikos C. Kyrpides, Hans-Peter Klenk

**Affiliations:** 1 DOE Joint Genome Institute, Walnut Creek, California, USA; 2 Los Alamos National Laboratory, Bioscience Division, Los Alamos, New Mexico, USA; 3 DSMZ – German Collection of Microorganisms and Cell Cultures GmbH, Braunschweig, Germany; 4 Biological Data Management and Technology Center, Lawrence Berkeley National Laboratory, Berkeley, California, USA; 5 Oak Ridge National Laboratory, Oak Ridge, Tennessee, USA; 6 HZI – Helmholtz Centre for Infection Research, Braunschweig, Germany; 7 University of California Davis Genome Center, Davis, California, USA

**Keywords:** dissimilatory nitrate-reducer, mesophile, free-living, marine, obligately anaerobic, motile, *Deferribacteraceae*, *Deferribacteres*, GEBA

## Abstract

*Denitrovibrio acetiphilus* Myhr and Torsvik 2000 is the type species of the genus *Denitrovibrio* in the bacterial family *Deferribacteraceae*. It is of phylogenetic interest because there are only six genera described in the family *Deferribacteraceae*. *D. acetiphilus* was isolated as a representative of a population reducing nitrate to ammonia in a laboratory column simulating the conditions in off-shore oil recovery fields. When nitrate was added to this column undesirable hydrogen sulfide production was stopped because the sulfate reducing populations were superseded by these nitrate reducing bacteria. Here we describe the features of this marine, mesophilic, obligately anaerobic organism respiring by nitrate reduction, together with the complete genome sequence, and annotation. This is the second complete genome sequence of the order *Deferribacterales* and the class *Deferribacteres,* which is the sole class in the phylum *Deferribacteres*. The 3,222,077 bp genome with its 3,034 protein-coding and 51 RNA genes is part of the *** G****enomic* *** E****ncyclopedia of* *** B****acteria and* *** A****rchaea * project.

## Introduction

**Figure 1:**
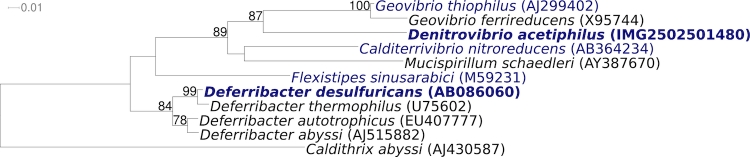
Phylogenetic tree highlighting the position of *D. acetiphilus* strain N2460^T^ relative to the other species within the phylum *Deferribacteres*. The tree was inferred from 1,460 aligned characters [3,4] of the 16S rRNA sequence under the maximum likelihood criterion [5] and rooted with *Caldithrix abyssi* ('Unclassified *Deferribacterales*'). The branches are scaled in terms of the expected number of substitutions per site. Numbers above branches are support values from 800 bootstrap replicates [6] if larger than 60%. Strains with a genome sequencing project registered in GOLD [7] are printed in blue; published genomes in bold [8].

Strain N2460^T^ (= DSM 12809) is the type strain of the species *Denitrovibrio acetiphilus*, which is the type species of the genus *Denitrovibrio* [1]. When this genus was described in 2000, it was the second validly published genus name in the phylum *Deferribacteres* Garrity and Holt 2001. Based on an extended analysis of 16S rRNA gene sequences, the phylum *Deferribacteres* was recently described as comprising the genera *Deferribacter, Denitrovibrio, Flexistipes, Geovibrio* and *Mucispirillum* [2]. However, the species *Calditerrivibrio nitroreducens* unequivocally also belongs to this phylum (Figure 1) [9].

In offshore oil extraction, reservoir souring by sulfate-reducing bacteria is of great economic concern. Seawater which naturally contains sulfates is injected into the reservoirs to enhance oil recovery. This sulfate load initiates the growth of sulfate-reducing bacteria producing H_2_S as the end product of sulfate respiration. Besides being toxic and corrosive, H_2_S increases the sulfur content of the oil and may contribute to the plugging of the reservoir [10,11]. Strain N2460^T^ was isolated from a laboratory model column simulating marine anoxic mineral oil reservoir conditions. The aim of these model experiments was to evaluate the feasibility to stop bacterial sulfate reduction by the addition of nitrate. The idea was to shift (redox) conditions in such a way that nitrate reducing populations supersede the sulfate-reducing populations. In the field, expensive biocides had often to be added to the injection water to prevent the negative effects of souring. For that reason, the application of nitrate or nitrite as a substitute showed great economic promise in oil exploitation [10]. There are several other older patents concerning the addition of nitrate or nitrite to aqueous systems with the aim to avoid biological H_2_S production and the associated odor nuisance (“Patent 4,681,687 cites the use of sodium nitrite to control SRB and H_2_S in flue gas desulfurization sludge”; US patent 5,405,531 of 1995 cites the injection of nitrate, nitrite and molybdate to inhibit sulfate reducing bacteria and hence prevent sulfide production). The application in order to manipulate the microbial communities in oil reservoirs has also been termed “Bio-Competitive Exclusion technology” [12].

In the laboratory model column from which strain N2460^T^ was isolated, bacterial sulfate reduction with crude oil as carbon and energy source was established first. Subsequently, the column was inoculated with an enrichment of nitrate-reducing bacteria deriving from ballast water, and 0.5 mM sodium nitrate was added to the circulating seawater [1]. Strain N2460^T^ was isolated after further enrichment in marine medium with acetate and nitrate as the electron donor and acceptor, respectively. As appraised by microscopic observation, the main population after nitrate application to the model column consisted of *Denitrovibrio acetiphilus*-like bacteria.

There are no reports of other strains of *D. acetiphilus* having been isolated. The species of the closest related genera, *Geovibrio* and *Deferribacter*, share 16S rRNA sequence identities of 85.3-85.9% and 84.2-85.7%, respectively [13]. The sequence similarity with phylotypes in environmental screenings and metagenomic libraries were all below 90%, except one single hit in the Wallaby gut metagenome (ADGC01007328, unpublished, 94%), indicating an extremely poor representation of closely related strains in the habitats analyzed (status March 2010). Here we present a summary classification and a set of features for *D. acetiphilus* strain N2460^T^, together with the description of the complete genome sequencing and annotation.

## Classification and features

Figure 1 shows the phylogenetic neighborhood of *D. acetiphilus* strain N2460^T^ in a 16S rRNA based tree. The two 16S rRNA gene sequences in the genome differ by one nucleotide from each other, and differ by up to one nucleotide from the previously published 16S rRNA sequence (AF146526) generated from DSM 12809.

**Figure 2:**
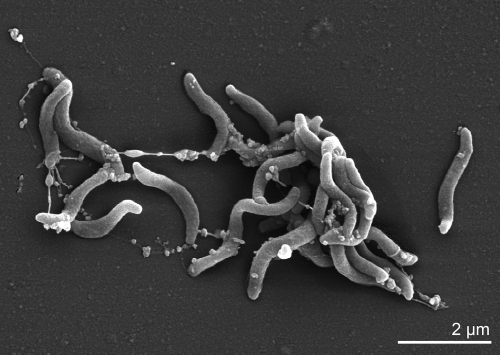
Scanning electron micrograph of *D. acetiphilus* strain N2460^T^

Cells of strain N2460^T^ are vibroid bacteria measuring 1.7-2.0 x 0.5-0.7 µm (Figure 2 and Table 1), multiplying by budding and showing rapid corkscrew movement. The strain is obligately anaerobic, and its growth is inhibited by oxygen and by anoxic non-reduced conditions. The bacterium is very versatile regarding the salt concentration of its environment as it grows in salt concentrations of 0 – 6% NaCl (w/v). It grows at temperatures between 4 and 40°C with an optimum at 35-37°C and at pH 6.5-8.6. The shortest doubling time at 35°C is about 8h. Vitamins are required for growth [1].

**Table 1:** Classification and general features of *D. acetiphilus* strain N2460^T^ according to the MIGS recommendations [14]

**MIGS ID**	**Property**	**Term**	**Evidence code**
	Classification	Domain *Bacteria*	TAS [15]
		Phylum *Deferribacteres*	TAS [16-18]
		Class *Deferribacteres*	TAS [16,19]
		Order *Deferribacterales*	TAS [16,19]
		Family *Deferribacteraceae*	TAS [16,20]
		Genus *Denitrovibrio*	TAS [1]
		Species *Denitrovibrio acetiphilus*	TAS [1]
		Type strain N2460	TAS [1]
	Gram stain	negative	TAS [1]
	Cell shape	vibroid	TAS [1]
	Motility	motile	TAS [1]
	Sporulation	spores not observed	TAS [1]
	Temperature range	mesophile, 4-40°C	TAS [1]
	Optimum temperature	35-37°C	TAS [1]
	Salinity	halophilic, grows at 0 - 6% (w/v) NaCl,	TAS [1]
MIGS-22	Oxygen requirement	obligately anaerobic, nitrate reducer	TAS [1]
	Carbon source	acetate or pyruvate (dissimilation) fumarate (fermentation)	TAS [1]
	Energy source	chemoorganotroph	TAS [1]
MIGS-6	Habitat	marine	TAS [1]
MIGS-15	Biotic relationship	free living	NAS
MIGS-14	Pathogenicity	none	NAS
	Biosafety level	1	TAS [21]
	Isolation	oil reservoir, model column	TAS [1]
MIGS-4	Geographic location	Bergen (Norway)	TAS [1]
MIGS-5	Sample collection time	about or before 2000	TAS [1]
MIGS-4.1 MIGS-4.2	Latitude Longitude	60.388 5.331	NAS
MIGS-4.3	Depth	unknown	
MIGS-4.4	Altitude	unknown	

Evidence codes - IDA: Inferred from Direct Assay (first time in publication); TAS: Traceable Author Statement (i.e., a direct report exists in the literature); NAS: Non-traceable Author Statement (i.e., not directly observed for the living, isolated sample, but based on a generally accepted property for the species, or anecdotal evidence). These evidence codes are from of the Gene Ontology project [22]. If the evidence code is IDA, then the property was directly observed by one of the authors or an expert mentioned in the acknowledgements.

Under the enrichment conditions, the cells gain energy by nitrate dissimilation with ammonia as the end product. In addition, the bacteria are able to grow on fumarate by fermentation [1]. The respiratory metabolism is restricted to a very limited substrate spectrum as the bacteria do not grow with benzoic acid, short chain alcohols, alkanes, carbohydrates, hydrogen or fatty acids other than acetate or pyruvate as the electron donor. However, this specialization on acetate needs not limit the spread of the organism in nature for acetate is a common fermentation product in almost any anoxic environment. As activity of 2-oxoglutarate dehydrogenase was present but carbon-monoxide dehydrogenase activity – the key-enzyme of the acetyl-CoA pathway –was absent in the cells, it was concluded that metabolization of acetate occurs via citric acid cycle [1].

As found for most strictly anaerobic nitrate reducing bacteria such as *Wolinella succinogenes* [23], *D. acetiphilus* reduces nitrate to the end product ammonia when growing by anaerobic respiration. This pathway should be delineated from the respiratory denitrification of facultatively anaerobic organisms which reduce nitrate to nitrous oxide or dinitrogen. Several obligately anaerobic nitrate-to-ammonium reducers gain energy only from the first reduction step from nitrate to nitrite (nitrate reductases). Some of these organisms may use this 6-electron transfer reduction as an electron sink for the regeneration of oxidized coenzymes during fermentation of carbohydrates, catalyzed by nitrite dependent reductase. In other anaerobes, such as *W. succinogenes, Desulfovibrio desulfuricans* or *D. gigas,* however, the reduction of nitrite to ammonia is also coupled to the electron transport phosphorylation [1]. Whether or not strain N2460^T^ is capable of gaining energy from the reduction of nitrite to ammonia is an unresolved question yet.

Another feature of the dissimilatory metabolism of strain N2460^T^ still awaits clarification: are these bacteria able to perform iron reduction as are several of its close phylogenetic relatives such as *Deferribacter thermophilus* or *Geovibrio ferrireducens*? Attempts to test for this ability in the lab failed because the addition of ferric pyrophosphate raised the redox potential to such an extend that growth of *D. acetiphilus*, which is sensitive to non-reduced conditions, was inhibited [1]. No other electron acceptor than nitrate (optimum concentration 8 mM) was found to support growth of strain N2460^T^ so far [1]. In this property, *D. acetiphilus* resembles another member of the *Deferribacteres, C. nitroreducens* which, however, is much more versatile regarding the electron donors than *D. acetiphilus* [9].

### Chemotaxonomy

Phospholipid fatty acids are the major fraction of the polar lipids contained in bacterial cells. The principal constituents of the phospholipids in N2460^T^ are unsaturated hexadecenoic acid and octadecenoic acid; other compounds are other straight chain saturated and unsaturated fatty acids [1]. The species *Flexistipes sinusarabici,* which also belongs to the phylum *Deferribacteres*, contains saturated hexadecanoic acid and octadecanoic acid as major compounds as well as iso- and anteiso-branched fatty acids in its polar lipids [1]. The predominant compounds in whole cell lipids of *C. nitroreducens* are iso-tetradecanoic and anteiso-pentadecanoic acid [9]. Thus, the yet described composition of the fatty acids within the *Deferribacteres* shows a wide variability. The presence of respiratory lipoquinones have not been reported, but it may be predicted that they should be present, since this is a feature of all members of the phylum examined to date.

## Genome sequencing and annotation information

### Genome project history

This organism was selected for sequencing on the basis of its phylogenetic position [24], and is part of the *** G****enomic* *** E****ncyclopedia of* *** B****acteria and* *** A****rchaea * project [25]. The genome project is deposited in the Genomes OnLine Database [7] and the complete genome sequence in GenBank. Sequencing, finishing and annotation were performed by the DOE Joint Genome Institute (JGI). A summary of the project information is shown in Table 2.

**Table 2:** Genome sequencing project information

**MIGS ID**	**Property**	**Term**
MIGS-31	Finishing quality	Finished
MIGS-28	Libraries used	Three genomic libraries: Sanger 8 kb, pMCL200 and fosmid libraries; one 454 pyrosequence standard library
MIGS-29	Sequencing platforms	ABI3730, 454 GS FLX
MIGS-31.2	Sequencing coverage	7.8× Sanger; 27.5× pyrosequence
MIGS-30	Assemblers	Newbler version 1.1.02.15, phrap
MIGS-32	Gene calling method	Prodigal 1.4, GenePRIMP
	Genbank ID	CP001968
	Genbank Date of Release	March 11, 2010
	GOLD ID	Gc01249
	NCBI project ID	29431
	Database: IMG-GEBA	2502422320
	Source material identifier	DSM 12809
	Project relevance	Tree of Life, GEBA

### Growth conditions and DNA isolation

*D. acetiphilus* strain N2460^T^, DSM 12809, was grown anaerobically in DSMZ medium 881 (*Denitrovibrio* medium) [26] at 30°C. DNA was isolated from 1-1.5 g of cell paste using Qiagen Genomic 500 DNA Kit (Qiagen, Hilden, Germany) with lysis modification st/L according to Wu *et al*. [25].

### Genome sequencing and assembly

The genome was sequenced using a combination of Sanger and 454 sequencing platforms. All general aspects of library construction and sequencing can be found at the JGI website (http://www.jgi.doe.gov/). Pyrosequencing reads were assembled using the Newbler assembler version 1.1.02.15 (Roche). Large Newbler contigs were broken into 3,494 overlapping fragments of 1,000 bp and entered into assembly as pseudo-reads. The sequences were assigned quality scores based on Newbler consensus q-scores with modifications to account for overlap redundancy and adjust inflated q-scores. A hybrid 454/Sanger assembly was made using the parallel phrap assembler (High Performance Software, LLC). Possible misassemblies were corrected with Dupfinisher or transposon bombing of bridging clones [27]. A total of 1,442 Sanger finishing reads were produced to close gaps, to resolve repetitive regions, and to raise the quality of the finished sequence. The final assembly contains 29,464 Sanger reads and 450,080 pyrosequencing reads. Together, the combination of the Sanger and 454 sequencing platforms provided 35.3× coverage of the genome. The error rate of the completed genome sequence is less than 1 in 100,000.

### Genome annotation

Genes were identified using Prodigal [28] as part of the Oak Ridge National Laboratory genome annotation pipeline, followed by a round of manual curation using the JGI GenePRIMP pipeline [29]. The predicted CDSs were translated and used to search the National Center for Biotechnology Information (NCBI) nonredundant database, UniProt, TIGR-Fam, Pfam, PRIAM, KEGG, COG, and InterPro databases. Additional gene prediction analysis and functional annotation was performed within the Integrated Microbial Genomes - Expert Review (IMG-ER) platform [30].

## Genome properties

**Figure 3:**
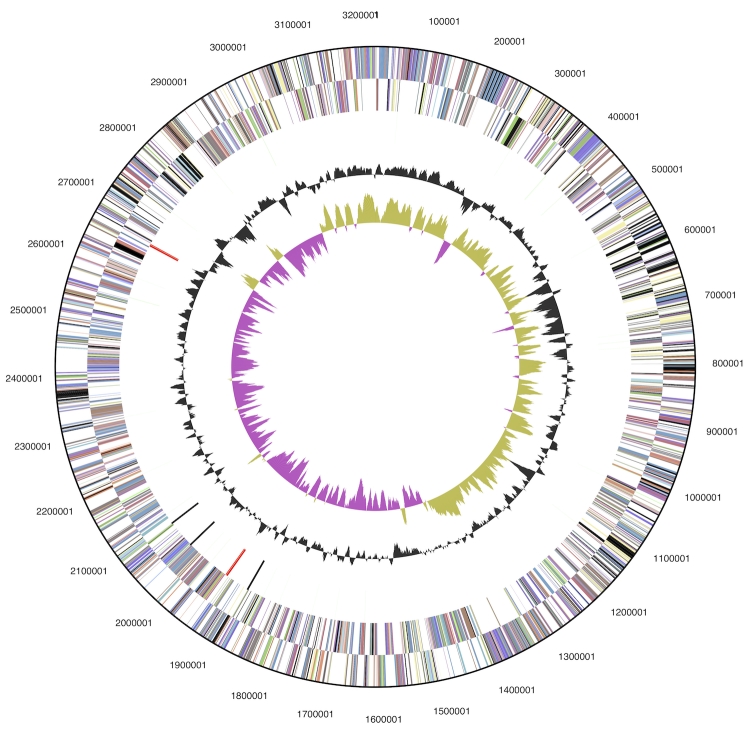
Graphical circular map of the chromosome. From outside to the center: Genes on forward strand (color by COG categories), Genes on reverse strand (color by COG categories), RNA genes (tRNAs green, rRNAs red, other RNAs black), GC content, GC skew.

The genome is 3,222,077 bp long and comprises one main circular chromosome with an overall G+C content of 42.5% (Table 3 and Figure 3) which is in very good accord with the figure given earlier after HPLC-determination (42.6%) [1]. Of the 3,085 genes predicted, 3,034 were protein-coding genes, and 51 RNAs; 70 pseudogenes were also identified. The majority of the protein-coding genes (74.4%) were assigned a putative function while those remaining were annotated as hypothetical proteins. The distribution of genes into COGs functional categories is presented in Table 4.

**Table 3:** Genome Statistics

**Attribute**	**Value**	**% of Total**
Genome size (bp)	3,222,077	100.00%
DNA coding region (bp)	3,006,341	93.30%
DNA G+C content (bp)	1,370,563	42.54%
Number of replicons	1	
Extrachromosomal elements	0	
Total genes	3,085	100.00%
RNA genes	51	1.65%
rRNA operons	2	
Protein-coding genes	3,034	98.35%
Pseudo genes	70	2.27%
Genes with function prediction	2,296	74.42%
Genes in paralog clusters	469	15.20%
Genes assigned to COGs	2,287	74.13%
Genes assigned Pfam domains	2,407	78.02%
Genes with signal peptides	620	20.10%
Genes with transmembrane helices	755	24.47%
CRISPR repeats	0	

**Table 4:** Number of genes associated with the general COG functional categories

**Code**	**value**	**%age**	**Description**
J	145	5.8	Translation, ribosomal structure and biogenesis
A	0	0.0	RNA processing and modification
K	147	5.8	Transcription
L	180	7.1	Replication, recombination and repair
B	1	0.0	Chromatin structure and dynamics
D	23	0.9	Cell cycle control, mitosis and meiosis
Y	0	0.0	Nuclear structure
V	46	1.8	Defense mechanisms
T	257	10.2	Signal transduction mechanisms
M	155	6.2	Cell wall/membrane/envelope biogenesis
N	103	4.1	Cell motility
Z	0	0.0	Cytoskeleton
W	0	0.0	Extracellular structures
U	74	2.9	Intracellular trafficking and secretion
O	89	3.5	Posttranslational modification, protein turnover, chaperones
C	220	8.7	Energy production and conversion
G	92	3.7	Carbohydrate transport and metabolism
E	182	7.2	Amino acid transport and metabolism
F	62	2.5	Nucleotide transport and metabolism
H	126	5.0	Coenzyme transport and metabolism
I	47	1.9	Lipid transport and metabolism
P	140	5.6	Inorganic ion transport and metabolism
Q	20	0.8	Secondary metabolites biosynthesis, transport and catabolism
R	263	10.4	General function prediction only
S	148	5.9	Function unknown
-	798	25.9	Not in COGs

## Insights in the genome

Anaerobic dissimilatory nitrate reduction can be carried out by denitrifying bacteria which are facultative anaerobes releasing the end product dinitrogen or by strict anaerobes which reduce nitrate to the end product ammonium. The first step, the reduction from nitrate to nitrite occurs in both metabolic types. The respective enzymes are encoded by gene families *nar* (nitrate reductase) and *nap* (periplasmic nitrate reductase) [31]. The operons encoding the nitrite reduction in denitrifying bacteria are named *nir, nor* and *nos* whereas the respective genes in the nitrate ammonifying bacteria are *nrf* [23]. The annotation of the N2460^T^ genome identified three genes encoding subunits of respiratory nitrate reductase (EC 1.7.99.4). These were identified as resembling known *narG, narH* and *narL* genes, thus they most probably encode for the alpha-, beta- and gamma-subunit of nitrate reductase. The automated search also detected  Dacet_0792 resembling in part the gene *nfrB* encoding for a compound of the multi-unit cytochrome c nitrite reductase.
